# Public procurement of medicines: scoping review of the scientific literature in South America

**DOI:** 10.1186/s40545-019-0195-9

**Published:** 2019-09-18

**Authors:** Cristiane Mota Soares, Beatriz Nascimento, Luisa Arueira Chaves, Rondineli Mendes Silva, Maria Auxiliadora Oliveira, Vera Lucia Luiza

**Affiliations:** 1 National School of Public Health Sergio Arouca, Oswaldo Cruz Foundation, Rio de Janeiro, RJ Brazil; 2 School of Pharmacy, Federal University of Rio de Janeiro, Macaé, RJ Brazil; 3 Department of Medicines and Pharmaceutical Services Policy, Sérgio Arouca National School of Public Health, Oswaldo Cruz Foundation, Rio de Janeiro, RJ Brazil

## Background

Access to medicines is essential for materializing access to health as a fundamental human right. As such, it is included in the United Nations Sustainable Development Goals [1], and is recognized as key-element for scaling-up access to health services towards universal health coverage [2]. Pharmaceutical services are not restricted to the logistical component of medicines availability. They also include management and quality of services, and promotion of adequate use of medicines. Even though, the availability of medicines is an extremely important dimension for the assurance of access to these products [3].

The scenario of intensification of technological innovations, the price of medicines has been a barrier to access, especially in low- and middle-income countries. In a context of expanding social policies in South America in the first decade of the twenty-first century, public procurement imposed negotiation processes and strategies to reduce the price of medicines.

Medicines have a major impact on governments, health care providers and household spending. Data from the Brazilian Health Satellite Account show that total government expenditures with medicines reached US$ 3,268,468 in 2015, representing a 54% increase over 2010 expenditures. Nevertheless, this represent around half of the household health expenditure, with a greater impact on the poorest families [4]. Barcelo et al. [5] estimated the per capita cost to treat diabetes mellitus in Latin America and the Caribbean (LAC) between US$ 1088 and US$ 1818 annually. In the same period, the average per capita of health care expenditure was US$ 1061 in LAC and medicines as a single item were responsible for a total of US$ 11–18 billion.

Availability is directly related to the procurement of medicines. Although there are different models and configurations of health systems, government procurement is an activity performed by all of them. It is as a tool for the fulfillment of public assignments, such as providing services to society, and its attainment is fundamental to the managerial and financial rationality of public administration. Government procurement is a type of public purchase carried out by entities linked to the State such as public companies and mixed-capital companies, and is governed by specific regulations of each country or region [6].

The difficult balance between best quality and low price has been a challenge in most countries. One reason for that is the lack of a standardized set of key-quality criteria for procurement monitoring and evaluation that promote transparency and improve governance. In order to contribute to reduce this gap, it is worth analyzing how the scientific community has focused on this theme. The present work aims to map the scientific production regarding processes of medicines public procurement in South American countries, with the objective of identifying main aspects discussed in peer-reviewed articles.

## Main text

### Methods

The method of scoping review systematized by Arksey and O’Malley [7] was used as tool for mapping scientific production, by including territorial and time variables. The present study covered 12 years, from 2005 - when it was held the second round of sub-regional antiretroviral (ARV) price negotiations in South America [8] - to 2017, of scientific production in articles published in English, Portuguese and Spanish in peer-reviewed journals. Given the importance of the above-mentioned price negotiation initiative, it is reasonable to expect that the event led to publication of studies in scientific journals.

### Research question

What is the scope and aspects discussed in the peer-reviewed articles on processes of medicines public procurement in South American countries?

### Data sources and search strategies

The search was carried out in the bibliographic databases SCOPUS, WEB OF SCIENCE (ISI), SCIELO, MEDLINE via PUBMED, EMBASE and Virtual Health Library (VHL), covering the fields of knowledge of health, social and human sciences. The Health Sciences Descriptors and keywords used in the study are listed in Table 1, with application of filtering per year of publication from 2005 to 2017.

**Table 1:** Search strategy for scientific publications on procurement of medicines in South America, syntax by database, from 2005 to 2017

Database	DeCS/ Keywords	Research date
VHL	ab:(“government purchasing” OR custos de medicamentos OR provisao OR suppl* OR purchas* OR “compra* governamenta* “OR “compra do* governo*” OR compra* OR aquisiç* OR provis* OR “processo de compra” OR “Proposta de Concorrência” OR provid* OR licitaç* OR procurement* OR bidding) AND (medicamento* OR drug* OR medicine* OR fármaco*) AND (brasil OR brazil OR venezuela OR argentina OR chile OR colombia OR suriname OR peru OR equador OR ecuador OR guiana OR guyana OR paraguai OR paraguay OR uruguai OR bolivia OR uruguay OR “South America” OR “america do sul”)	10/30/2017
Scopus	((TITLE-ABS-KEY (drug*) OR TITLE-ABS-KEY (medicine*) OR TITLE-ABS-KEY (drug costs) OR TITLE-ABS-KEY (“Pharmaceutical Preparations”))) AND (((TITLE-ABS-KEY (“government purchasing”) OR TITLE-ABS-KEY (suppl*) OR TITLE-ABS-KEY (purchas*) OR TITLE-ABS-KEY (provid*) OR TITLE-ABS-KEY (procurement*) OR TITLE-ABS-KEY (bidding))) AND (TITLE-ABS-KEY (brazil OR venezuela OR argentina OR chile OR colombia OR suriname OR bolivia OR peru OR ecuador OR guyana OR paraguay OR uruguay OR “South American”))	10/31//2017
Pubmed via Medline	((brazil OR venezuela OR argentina OR chile OR colombia OR suriname OR peru OR ecuador OR guyana OR bolivia OR paraguay OR uruguay OR “South American”)) AND ((((((“pharmaceutical preparations”[MeSH Terms]) OR “drugs”[Title/Abstract]) OR “medicines”[Title/Abstract])) AND (((((“purchasing”[Title/Abstract]) OR “group purchasing”[MeSH Terms]) OR ((“purchasing health care”[Title/Abstract] OR “purchasing health insurance”[Title/Abstract] OR “purchasing health services”[Title/Abstract] OR “purchasing medication”[Title/Abstract] OR “purchasing medications”[Title/Abstract] OR “purchasing medicine”[Title/Abstract] OR “purchasing medicines”[Title/Abstract]))) OR “drug purchasing”[Title/Abstract]) OR “medicines supply”[Title/Abstract])))	10/31/2017
Web of science	TS = ((“government purchasing” OR suppl* OR drug costs OR purchas* OR provid* OR procurement* OR bidding) AND (Brazil OR venezuela OR argentina OR chile OR colombia OR suriname OR peru OR ecuador OR guiana OR guyana OR paraguai OR paraguay OR uruguay OR bolivia OR South America))	11/05/2017
Embase	(‘drug’:ti,ab,kw OR ‘medicine’:ti,ab,kw OR ‘pharmaceutics’:ti,ab,kw) AND (‘supply’:ti,ab,kw AND ‘distribution’:ti,ab,kw OR ‘provide’:ti,ab,kw OR ‘procurement’:ti,ab,kw OR ‘bidding’:ti,ab,kw OR ‘supply’:ti,ab,kw OR drug costs: ti,ab,kw) AND (‘brazil’:ti,ab,kw OR ‘argentina’:ti,ab,kw OR ‘colombia’:ti,ab,kw OR ‘venezuela’:ti,ab,kw OR ‘bolivia’:ti,ab,kw OR ‘guyana’:ti,ab,kw OR ‘uruguay’:ti,ab,kw OR ‘paraguay’:ti,ab,kw OR ‘peru’:ti,ab,kw OR ‘south america’:ti,ab,kw OR ‘ecuador’:ti,ab,kw OR ‘suriname’:ti,ab,kw OR ‘chile’:ti,ab,kw)	11/17/2017
Scielo	(medicamento* OR medicine OR drug OR prepara* farmaceutic* OR custos de medicamentos) AND (compra OR supply OR purshasing OR procurement OR provide OR provis* OR licita* OR bidding OR aquisi* OR drug costs) AND (brazil OR brasil OR argentina OR venezuela OR chile OR peru OR “america do sul” OR “south america” OR Guyana OR uruguai OR uruguay OR guiana OR colombia OR bolivia OR suriname OR paraguai OR paraguay OR equador OR ecuador)	11/17/2017

The selection of articles and extraction of data were performed independently by two of the authors; a third author resolved disagreements.

### Eligibility criteria

The publications were selected when they focused on the procurement of medicines for public purposes and included at least one South American country at the regional, national and subnational levels. Consortium purchase cases were included regardless of population size, as well as studies of government expenditures and international price comparisons, as long as they included the public sector. It was decided to use the term “public purchase” to cover also the purchases made by private entities and non-profit organizations, provided these were done for public purposes. Finally, we retrieved full text articles available in the Periodicos CAPES database as well as in open-access journals.

Papers addressing private purchases, or specific institutions such as hospitals, or Brazilian municipalities with less than 500,000 inhabitants were excluded because they represent local level processes. We also excluded publications of bibliographic review, development of methodologies, editorials and essays. Cost-effectiveness studies, in which the purchase process was not the main focus because they only assessed medicines prices were also excluded.

### Data summary and synthesis

The following data were extracted from the selected articles: year of publication, country and institution of the first author, language of publication, study population, period studied (year), study design, main results and conclusion. The studies were categorized according to study design into: partial health economic evaluation, qualitative method, and mixed-methods. The definition of partial health economic evaluation (PHEE) updated by Drummond et al. [9] was adopted. When qualitative approach was combined with PHEE it was categorize as mixed-method.

The studies were also categorized according to the approach and themes approached as: pooled purchasing; public purchasing profile; medicines prices, availability, and affordability - World Health Organization (WHO)/ Health Action International (HAI) methodology - and international mechanisms for medicines procurement.

## Results

**Fig. 1:**
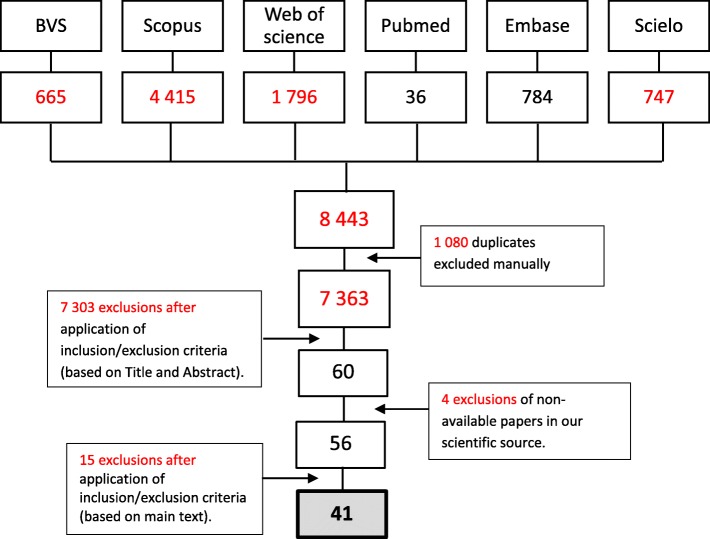
Stepwise process for selection of papers concerning procurement of medicines in South America from 2005 to 2017

In total, 8443 references were obtained. After exclusion of duplicates and application of the selection criteria, 41 articles were kept (Fig. 1). Of these, 46.3% were published in English only, 31.7% in English and Portuguese, 14.6% in Portuguese only, and 7.4% in Spanish.

Most articles (53.7%) focused on a single country and 22% studied a number of countries, ranging from 6 to 52 countries, which often included countries from other regions outside South America. Articles about Brazil approached purchases of a single municipality (12.2%), of a number of municipalities by consortium (4.9%), of the general population (2.4%) and two articles evaluated Brazilian state level purchase (4.9%).

**Fig. 2:**
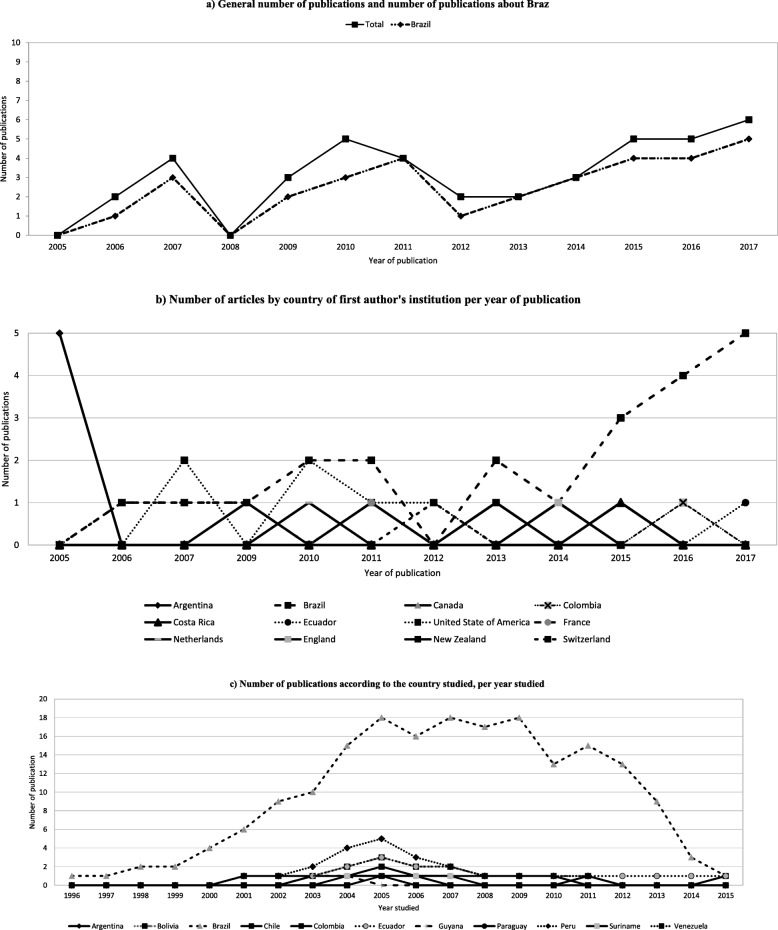
Number of articles according to selected variables from 2005 to 2017. **a** General number of publications and number of publications about Brazil. **b** Number of articles by country of first author’s institution per year of publication. **c** Number of publications according to the country studied, per year studied

Researchers from Brazil produced more than half of the selected publications (51.2%). Other countries that contributed to the academic production on the studied theme were the United States of America (14.6%), Switzerland (9.8%), and Argentina (4.9%). During the time interval reviewed, there was a general increasing in publications on the theme, with a decrease in 2008 and a later resumption, as shown in Fig. 2a. In recent years this increase was leveraged by publications conducted by Brazil as first author residence country (Fig. 2b).

Teaching/research institutions produced 73.2% of the papers. Productions from governmental bodies such as Health Ministries and Secretariats accounted for 12.2% of the papers. International organizations, such as WHO, presented an equivalent proportion. Data considered on the selected studies covered from 1996 to 2015, with a peak in 2007 and 2009 in the studies concerning Brazil (Fig. 2c). The most studied country was Brazil (78%), followed by Peru (17.1%) and Ecuador (12.2%).

Most of the selected studies (82.9%) applied the HEE method. Qualitative approach corresponded to 7.3%, and the mixed method was the strategy used in 9.8% of the papers. The data were summarized in Table 2.

**Table 2:** Systematization of articles by year of publication, country of publication, time covered, countries covered and study designer

*Reference*	*Year of publication*	*Country of publication*	*Time covered*	*Countries covered*	*study designer*
*Pooled purchasing*					
[10]	2011	Brazil	2007–2009	Brazil	PHEE
[11]	2007	Brazil	2000	Brazil	PHEE
*Public purchasing profile*					
[12]	2016	Brazil	2012	Brazil	PHEE
[13]	2014	Brazil	2011	Brazil	PHEE
[14]	2009	Argentina	2005	Argentina	PHEE
[15]	2017	Brazil	2012	Brazil	PHEE
[16]	2010	USA	2003–2004	Guiana	PHEE /QR
[17]	2016	Brazil	2008–2014	Brazil	PHEE
[18]	2014	Brazil	2002–2011	Brazil	PHEE
[19]	2014	Brazil	2004–2011	Brazil	PHEE
[20]	2015	Brazil	2008–2013	Brazil	PHEE
[21]	2016	Brazil	2004–2013	Brazil	PHEE
[22]	2007	USA	2001–2005	Brazil	PHEE
[23]	2011	France	1996–2009	Brazil	PHEE
[24]	2011	Brazil	2009	Brazil	PHEE
[25]	2017	Brazil	2004–2013	Brazil	PHEE
[26]	2015	Costa Rica	2001–2006	Chile	PHEE
[27]	2016	Colombia	2015	Colombia	PHEE
[28]	2013	Brazil	2005–2009	Brazil	PHEE
[29]	2009	Brazil	2002–2007	Brazil	PHEE
[30]	2015	Brazil	200–2012	Brazil	PHEE/ QR
[31]	2017	Ecuador	2012–2015	Ecuador	QR
[32]	2017	Brazil	2006–2013	Brazil	PHEE
[33]	2017	Brazil	2007–2014	Brazil	PHEE
[34]	2006	Brazil	1998–2008	Brazil	PHEE
[35]	2015	Brazil	2005–2013	Brazil	PHEE
[36]	2016	Brazil	2001–2012	Brazil	PHEE
[37]	2017	Brazil	2005–2015	Brazil	PHEE
*Comparison of prices, availability, and affordability (WHO/HAI methodology)*					
[38]	2007	Switzerland	2005	Brazil	PHEE
[39]	2010	Netherlands	2004	Brazil and Peru	PHEE
[40]	2010	USA	2005	Peru	PHEE
[41]	2010	Brazil	2007	Brazil	PHEE
[42]	2009	Switzerland	2001–2006	Brazil and Peru	PHEE
[43]	2012	Switzerland	2003–2010	Bolivia, Ecuador, Colombia, Peru and Brazil	PHEE
[44]	2013	New Zealand	2011	Brazil, Chile, Ecuador and Peru	PHEE
*International mechanisms for purchase of medicines*					
[45]	2006	Switzerland	2004–2005	Uninformed	PHEE
[46]	2007	USA	Uninformed	Chile, Colombia, Ecuador, Peru, Venezuela and Bolivia	QR
[47]	2010	Brazil	2004–2007	Bolivia, Brazil, Ecuador, Paraguay, Peru and Suriname	PHEE /QR
[48]	2011	Argentina	Uninformed	Argentina, Brazil, Paraguay, Uruguay and Venezuela	QR
[49]	2012	USA	Uninformed	Uninformed	QR
[50]	2015	Canada	2002–2013	Brazil	PHEE

*USA* United States of America, *PHEE* Partial Health Economic Evaluation, *QR* Qualitative research

Almost a quarter (21.9%) of the papers primarily addressed ARV medicines for HIV/AIDS treatment. The articles were distributed into four analytical categories.

### Pooled purchasing

Two articles analyzed purchases of municipalities by consortium in two states in the southern region of Brazil [10, 11]. Amaral and Blatt [10] undertook a comparative study of purchases of the municipality of Indaia from 2007 to 2009 and observed a positive effect in reducing medicines shortages and prices, by consortium purchase among municipalities. This reduction of prices is in line with data published by Ferraes and Cordoni Júnior [11] in an analysis of a consortium in the state of Paraná involving 352 municipalities in the year 2000, a strategy that resulted in an average price approximately 30% lower than the values ​​of the Ministry of Health Prices Database.

### Public purchasing profile

The purchasing profile was analyzed in 26 articles restricted to purchases by government agencies, of which 21 were related to federal entities - including six surveys with a comparative perspective among subnational entities. Only five articles discussed exclusively municipal purchases, and of these, three were from Rio de Janeiro, Brazil [12, 17, 18], one was from São Paulo, Brazil [13], and one was from Rosario, Argentina [14]. All articles produced by government institutions - Ministries and Secretariats of Health - and the majority (66.7%) of articles produced by research/teaching institutions were included in this category of analysis.

Armijos et al. [31] made a documentary research to analyze the role of Health Technology Assessment in the processes of purchase of medicines that were not included in the Ecuador National List of Essential Medicines.

The use of information technology tools such as electronic purchase showed results in reducing the price of medicines when evaluated in the context of Chile in 2004 [26].

In the analysis of federal purchases in the period 2004 to 2013, Teodoro et al. [25] observed low, but growing rates of acquisition of medicines without commercialization approval in Brazil. These purchases were justified by judicial demand in 82% of the cases. The judicial claim as reasoning for the purchase of medicines by government agencies is a topic discussed in four studies [13, 19–21]. These studies addressed the economic impact, the potential effects on the incorporation of new technologies [22, 23], and responsibilities of the federative entities in medicines acquisition and distribution.

The costs of public pharmaceutical services, in the context of the Rio de Janeiro municipality [12] and Minas Gerais state - Pharmacy Network of Minas Program [15] - were compared to the public Brazilian program called Popular Pharmacy. Historical series of expenditures of federated entities in Brazil [24, 28] and comparisons between specifics programs [29] provided an overview of public purchase. Some papers focused on the organization of public financing, as well as the acquisition and distribution chain in Brazil [30] and in other countries such as Guyana [16]. Price comparison of generic oncological medicines was central in an article from Colombia [27].

The characterization of purchases involved data collection on medicine type, dose, volume of the purchase, unit price, date, reasoning of the purchase, and type of acquisition process including a diverse range of medicines [14, 17–19, 32–34] or a therapeutic class [20, 21, 35–37], with cost per capita estimation and, in some cases, comparison with international prices or prices of specific partnerships [35].

Statistical analyses and mathematical models were adopted for different purposes. The Pareto curve for municipal purchases [14] considered the importance based on the quantities purchased and the expense. The Lorenz curve, used in the article by Luz et al. [32], allowed to discuss whether the distribution of the purchased volume per therapeutic group was equitable.

Regarding Brazil, some studies used the Integrated System for General Services Administration to obtain public procurement data. This web-based system has data from the Federal Government, but also from providers and institutions that use the *Comprasnet* system. Thus, some articles made a combined analysis of the federal with purchases made by the state and municipal health departments [20, 24, 28, 30]. The *Comprasnet* system allow the realization of electronic procurement processes and make available to society, information regarding the bids and hires promoted by the Federal Government.

The Information System on Public Health Budget allowed for comparison of expenditures by subnational levels, constituting a source of public procurement data for states, the federal district, and Brazilian municipalities. Another data sources in Brazil are the Price Record Minutes and the federal, state and municipal Official Gazette. The Access to Information Act allows requesting data not easily available. The Colombia Information System on Medicines Prices was used in a study comparing the governmental purchasing prices with generic medicines prices available in the Colombian market [27].

Most articles in this category referred to Brazil (80.8%) [12, 13, 15, 17–25, 28–30, 32–37] but they also included Argentina [14], Guyana [16], Chile, Colombia [26, 27] and Ecuador [31].

### Comparison of prices, availability, and affordability (WHO/HAI methodology)

The international price comparison was the subject of seven surveys that applied the WHO/HAI methodology. This methodology is often used to assess prices, and availability, and affordability, and cost of a set of medicines in countries, and compares prices between public and private sectors.

Most of the studies that used the WHO/HAI methodology compared prices of medicine therapeutic groups such as: antiepileptics [43]; drugs for cardiovascular diseases [39]; drugs for asthma [44]; and antihypertensive and antidiabetic agents [41]; and more general for chronic diseases [38].

One study compared prices, availability, and affordability of a set of 15 medicines in 36 developing countries [42]; another study assessed the adequacy of the target WHO/HAI medicines list and sampling strategy in Peru [40]. The study applied the WHO/HAI methodology in Peru in 2005 considering remote areas and demonstrated an appropriate balance between modest research costs and optimal information for policy.

In general, the publications covered Brazil [38, 39, 41–44], Peru [39, 40, 42–44], Bolivia [43], Ecuador [43, 44], Colombia [43] and Chile [44].

### International mechanisms for purchase of medicines

Six articles analyzed international mechanisms for medicines price negotiation and procurement, such as the Pan American Health Organization (PAHO) Strategic Fund, joint negotiation for high-cost medicines among groups of countries (MERCOSUR and Andean Community), and purchases through the Global Fund to Fight HIV/AIDS, Global Drug Facility (GDF) to Tuberculosis (TB) and Malaria. The Global Drug Facility (GDF) is housed and administered by WHO. The Southern Common Market (MERCOSUR for its Spanish initials) is a regional integration process, initially established by Argentina, Brazil, Paraguay and Uruguay, and subsequently joined by Venezuela and Bolivia. The Andean Community includes Bolivia, Colombia, Ecuador and Peru.

One study made more qualitative analyses of the processes of international joint procurement mechanisms [49], seeking for analyzing the potential of joint purchases to reduce costs, achieve economies of scale, improve rational therapeutic choices and reduce counterfeit medicines.

Regarding funding, Vasan et al. [45] used the 2004 Purchase Price Report, released in by the Global Fund to Fight AIDS, Tuberculosis, and Malaria, to analyze the acquisition process of the main GDF subside receivers. They observed that persistently high prices for ARV medicines continue to slow the spreading of HIV/AIDS treatment in developing countries. Moreover, the authors identified that there are many divergences in the procurement process and pricing of ARV drugs. This indicates the importance of ensuring the transparence of ARV acquisition data in order to reduce such discrepancies, that was discuss in other paper [50]. A strong base of evidence about prices could enable developing countries to make less costly procurement choices.

Another paper [47] addressed the PAHO Strategic Fund created in 2000 to improve procurement of essential drugs for HIV/AIDS, tuberculosis, malaria and leishmaniasis treatment. This research described the operation of the Fund as well as its procurement activities from 2004 to 2007. The Fund mobilized approximately US$ 3 million to US$ 19 million in the period studied. From January to September 2007, Brazil accounted for 63% of expenditures on strategic health supplies, corresponding to approximately US$ 12 million.

Regarding multi-countries joint negotiation strategies, Seoane-Vazquez and Rodriguez-Monguio [46] evaluated the price negotiation processes between Andean Community and the pharmaceutical industry. In this regard, the article analyzed problems faced by this group of countries during joint negotiation process, and the factors that would have hampered the implementation of the negotiated prices by the countries.

Marín and Polach [48] examined how Mercosur countries managed to access, regulate and pay for high-cost medicines and proposed strategies for joint selection and financing at the sub-regional level.

## Discussion

The focus of most of the studies analyzed was the Brazilian system. This country was a pioneer in the scientific production on public procurement in South America. International price comparisons were also widely adopted involving other countries.

Price comparison were broadly assessed in the studies as the main expression of procurement or negotiation success. Indeed, the lowest unit medicine price was the most mentioned measure of efficiency. Other medicines procurement process components such as delivery time, purchase type, medicine quality and logistic support were not focused, which means a scientific gap on those issues.

The WHO/HAI methodology has been widely used in studies, especially those comparing prices and availability of medicines in different countries. This methodology is validated [40] and internationally accepted to produce reliably evidence. Moreover, it reduces survey costs and allows for greater comparability of results in different countries.

The analysis of international mechanisms for negotiating prices and purchasing medicines indicates that the studies in different countries have shown reliable results, especially on possibilities of price reductions and gains in scale. However, the sustainability of these mechanisms in the medium term is questionable, especially because countries often fail to complete the purchase process after price negotiation, often due to issues related to internal regulation [8].

The low diversity of studies in terms of territorial coverage makes it difficult to know the similarities and differences among South American countries. However, Marin & Polach [48] brought important comparisons on the structure and organization of health systems in terms of medicines purchases, among the MERCOSUR countries, with the objective to propose mechanisms to improve the purchase of high-cost medicines.

The great majority of the articles were published in English, despite all studies included addressed South American countries. This may indicate authors’ effort to increase the diffusion of knowledge.

Research and teaching institutions played a leading role, consistent with the mission of this sector, but research has also been carried out within the framework of important international organizations - WHO and PAHO - and governmental organizations - Secretariats and Ministries of Health. The focus of the studies carried out by government organizations on purchase profile surveys was related to concerns regarding drug prices and procurement policies comparisons. The focus of studies carried out by international organizations targeted joint procurement mechanisms, what is related to analysis of funds and comparative perspective of medicines prices.

Analysis of public purchases profile have been proven useful for several purposes and constitute a proxy for the use of medicines. They have been important to measure the degree of implementation of the National Lists of Essential Medicines over time, as well as the development of public policies [32].

Although considered by two studies [12, 21], the issue of costs involved in purchase processes is barely discussed despite its great importance for the comparison of government programs. These costs include the payment of professionals and logistics for acquisition among other elements. As indicated by the articles analyzed here, this is an important aspect not explained by the simple comparison of the final prices obtained. Likewise, the level of government taxation may vary among different federal entities, as in the case of Brazil, and this may have important implications.

ARV medicines were the focus of a couple of studies that contextualize purchases in the period from 1996 to 2016. These studies included issues related to the scale-up of access to ARV medicines, changes in the incorporation of new ARV, negotiation strategies, compulsory licensing to allow for local production, purchase via international mechanisms, among other actions. Historical series of purchases represent the method adopted in some researches that analyze public health policy. Other research methods such as interviews and documentary analysis are also used.

Purchase analysis data mainly comprise federal expenditures, either through the Federal level’s role as coordinator of the health policy - even in decentralization health systems - or by the possible facilitated access to the information system. The scientific production of government institutions - Health Ministries and Secretariats - at federal and municipality level, focuses on the profile of purchases and spending on medicines, as well as the comparison between programs and federated entities.

Increases in drug spending, quantity and diversity were identified in several studies, which could indicate an increased availability in cases of high prices of new medicines.

In general, the partial economic health surveys discussed the economic and budgetary sustainability impacts, without losing focus of the right to health and the need to increase access to medicines. These studies aim to improve the rationality of expenses and possibilities of coping with high prices medicines.

### Study limitations

The intention of this study was to capture the literature placed in dialogue with the scientific community, after peer review. Having the search conducted in October 2017, articles indexed this month or later, were not included in this review.

The terms applied in the searches were the most comprehensive as possible. However, given the extensive universe of publications, it was decided to include the names of the countries and the sub-region were included as a filter to enable the selection stage. Thus, studies that did not explicitly used these terms in the fields searched may not have been retrieved.

## Conclusions

Despite the broad scope of the selected papers, there are important aspects of the purchase process neglected in scientific production, which would have contributed to a better understanding of the purchase process undertaken in different South American countries. Market dynamics, the time for execution of the process, and the deepening of the description of suppliers and/or manufacturers, as well as the elements that involve the types of purchase processes, were little discussed themes.

All South American countries were addressed in at least one study; this result seems to be influenced by the application of the method developed by the WHO/HAI, showing its relevance to enable the production of information on the theme of availability and price per country, as well as the dissemination of this information on scientific publications.

The scoping review allowed an overview of the scientific production of public procurement of medicines within a space-time perspective, involving the issues that permeate public purchases field of knowledge and the methods applied to reach the objectives established by the studies. This review discusses this production, to widening the debate, opening possibilities for partnerships, and indicating knowledge gaps.

## Data Availability

The datasets used and/or analysed during the current study are available from the corresponding author on reasonable request.

## Abbreviations

ARV: antiretroviral
GDF: Global Fund to Fight HIV/AIDS, Tuberculosis and Malaria
HAI: Health Action International
HEE: health economic evaluation
LAC: Latin America and the Caribbean
PAHO: Pan American Health Organization
PHEE: Partial Health Economic Evaluation
USA: United States of America
VHL: Virtual Health Library
WHO: World Health Organization
