# Correction to: Public procurement of medicines: scoping review of the scientific literature in South America

**DOI:** 10.1186/s40545-019-0197-7

**Published:** 2019-11-18

**Authors:** Cristiane Mota Soares, Beatriz Nascimento, Luisa Arueira Chaves, Rondineli Mendes Silva, Maria Auxiliadora Oliveira, Vera Lucia Luiza

**Affiliations:** 1 National School of Public Health Sergio Arouca, Oswaldo Cruz Foundation, Rio de Janeiro, RJ Brazil; 2 School of Pharmacy, Federal University of Rio de Janeiro, Macaé, RJ Brazil; 3 Department of Medicines and Pharmaceutical Services Policy, Sérgio Arouca National School of Public Health, Oswaldo Cruz Foundation, Rio de Janeiro, RJ Brazil

**Correction to: J Pharm Policy Pract (2019) 12:33**

**https://doi.org/10.1186/s40545-019-0195-9**

Following publication of the original article [1], we have been notified of a few errors in the figures:

- Figure 1: the red text needs to be black and not red
- Figure 2: In 2005 the publication number for Argentina was 0 (zero) and not 5.

The original article has been corrected.
